# Hereditary Transmission of Mutilations

**Published:** 1892-10

**Authors:** 


### Hereditary Transmission of Mutilations.

Dr. C. G. Lockwood, of New York, has recently published some interesting results of his experiments on the hereditary transmission of multilations. White mice were selected on account of their rapid breeding, as they begin to breed every thirty days. He bred them in-and-in for ninety-six generations, destroying all the sickly and defective ones, and in this way obtained a larger and finer animal than the original pair. His experiments in breeding their tails off were done by selecting a pair and putting them in a cage by themselves and clipping the tails of all the young. When these were old enough to breed he selected a pair, and when they had young, clipped their tails. By continuing this breeding, in the seventh generation he got some young without tails, and finally got a perfect breed of tailless mice. By taking one with a tail and one without a tail, and alternating the sexes in each generation, he finally again got a breed of all-tail mice.—*N. Y. Med. Record.*

